# The effect of therapeutic drug monitoring of beta-lactam and fluoroquinolones on clinical outcome in critically ill patients: the DOLPHIN trial protocol of a multi-centre randomised controlled trial

**DOI:** 10.1186/s12879-020-4781-x

**Published:** 2020-01-17

**Authors:** A. Abdulla, T. M. J. Ewoldt, N. G. M. Hunfeld, A. E. Muller, W. J. R. Rietdijk, S. Polinder, T. van Gelder, H. Endeman, B. C. P. Koch

**Affiliations:** 1 Department of Hospital Pharmacy, Erasmus University Medical Center, P.O. Box 2040, 3000 CA Rotterdam, the Netherlands; 2 Department of Intensive Care, Erasmus University Medical Center, Rotterdam, The Netherlands; 3 Department of Medical Microbiology and Infectious Diseases, Erasmus University Medical Center, Rotterdam, The Netherlands; 4 Department of Medical Microbiology, Haaglanden Medical Center, The Hague, The Netherlands; 5 Department of Public Health, Erasmus University Medical Center, Rotterdam, The Netherlands; 6 Department of Internal Medicine, Erasmus University Medical Center, Rotterdam, The Netherlands

**Keywords:** Randomised controlled trial, Therapeutic drug monitoring, Antibiotic, Beta-lactam, Fluoroquinolones, Critically ill patients, Length of stay, Pharmacokinetics, Pharmacodynamics

## Abstract

**Background:**

Critically ill patients undergo extensive physiological alterations that will have impact on antibiotic pharmacokinetics. Up to 60% of intensive care unit (ICU) patients meet the pharmacodynamic targets of beta-lactam antibiotics, with only 30% in fluoroquinolones. Not reaching these targets might increase the chance of therapeutic failure, resulting in increased mortality and morbidity, and antibiotic resistance. The DOLPHIN trial was designed to demonstrate the added value of therapeutic drug monitoring (TDM) of beta-lactam and fluoroquinolones in critically ill patients in the ICU.

**Methods:**

A multi-centre, randomised controlled trial (RCT) was designed to assess the efficacy and cost-effectiveness of model-based TDM of beta-lactam and fluoroquinolones. Four hundred fifty patients will be included within 24 months after start of inclusion. Eligible patients will be randomly allocated to either study group: the intervention group (active TDM) or the control group (non-TDM). In the intervention group dose adjustment of the study antibiotics (cefotaxime, ceftazidime, ceftriaxone, cefuroxime, amoxicillin, amoxicillin with clavulanic acid, flucloxacillin, piperacillin with tazobactam, meropenem, and ciprofloxacin) on day 1, 3, and 5 is performed based upon TDM with a Bayesian model. The primary outcome will be ICU length of stay. Other outcomes amongst all survival, disease severity, safety, quality of life after ICU discharge, and cost effectiveness will be included.

**Discussion:**

No trial has investigated the effect of early TDM of beta-lactam and fluoroquinolones on clinical outcome in critically ill patients. The findings from the DOLPHIN trial will possibly lead to new insights in clinical management of critically ill patients receiving antibiotics. In short, to TDM or not to TDM?

**Trial registration:**

EudraCT number: 2017–004677-14. Sponsor protocol name: DOLPHIN. Registered 6 March 2018 . Protocol Version 6, Protocol date: 27 November 2019.

## Background

In intensive care units (ICU) critically ill patients from all medical specialties are treated. Consequently, the ICU population is highly heterogeneous and among the most complex and expensive within healthcare [1]. Results of a large international prospective trial show that 70% of ICU patients receive antibiotics [2]. However, both the incidence of infections and associated mortality in the ICU have not improved over the last 30 years [3–5]. This indicates that improvements in clinical outcomes of ICU patients might be possible.

Standard dosing regimens of antibiotics are usually empirically prescribed based upon a most probable diagnosis. Due to physiological changes in ICU patients, the pharmacokinetic (PK) behaviour is different from non-ICU patients and subject to impressive changes. Augmented renal clearance is prevalent, even with normal serum creatinine levels [6, 7]. Dosing regimens used are designed for non-severely ill patients and derived from studies in healthy volunteers. This might result in inadequate antibiotic treatment in critically ill patients. Frequent changes in the renal function, volume of distribution and extravascular loss of fluids are also prevalent, which results in pharmacodynamic variability [8]. Furthermore, parameters frequently used in patients on the regular wards, such as serum creatinine, might not be reliable in ICU patients. As a consequence, this results in suboptimal dosing followed by treatment failure and increased mortality [9].

Dosing of antibiotics is based upon the minimum inhibitory concentration (MIC) of micro-organisms. The actual MIC is often unknown and unreliable to determine [10]. The epidemiological cut-off values (ECOFF) describes, for a given species and antibiotic, the highest MIC for organisms devoid of phenotypically-detectable acquired resistance mechanisms. It defines the upper end of the wild-type distribution [11]. Pharmacokinetic/pharmacodynamic (PK/PD) relationships have been described for most antimicrobial classes. These relationships show a marked consistency, and the pharmacodynamic index values that result in a certain effect have been determined for most classes of antibiotics [12]. The pharmacodynamic targets (PDTs) are the minimum value of the PK/PD index that are based on preclinical and clinical drug/micro-organism exposure-response relationships.

Not reaching antibiotic PDTs is associated with therapeutic failure and increased microbial resistance [13–15]. Target attainment is reported only in 60% of beta-lactam use in the ICU [16]. Ciprofloxacin, a fluoroquinolone has a reported target attainment of respectively 60–80%, and 17–30% for bacteria with MICs of ≤0.25, and 0.5 mg/L [17–20]. As imaginable, therapeutic failure might increase ICU length of stay (ICU LOS). Prolonged ICU LOS is associated with higher ICU, hospital, and 1-year mortality rate [21] as well as greater use of ICU resources [22]. On the other hand, high dosing regimens might result in trough levels associated with toxicity [23]. Simply increasing the standard dosing on the ICU is therefore not optimal: the inter- and intrapatient variability is too high.

Therapeutic Drug Monitoring (TDM) might be used to optimise pharmacological target attainment and therefore decrease therapeutic failure [24]. Dose adjustments will need to be made in an early phase of treatment, since quick intervention in antibiotics is essential for patients with sepsis [25]. Usually a trough concentration (C_trough_) is used for asserting antibiotic effectiveness. However steady state trough concentrations may not be reached before four previous doses of medication [26]. To predict those concentrations, model based TDM for individualized therapy might be a valuable tool [27].

To the best of our knowledge, no RCT has investigated the effect of TDM of beta-lactam and fluoroquinolones on clinical outcomes in critically ill patients.

### Primary objective

The primary objective of the trial is to determine the effect of early model-based TDM of beta-lactam and fluoroquinolones on clinical outcome in critically ill patients.

## Methods and design

The DOLPHIN trial is a prospective, multi-centre, RCT investigating whether early model-based therapeutic drug monitoring of beta-lactam and fluoroquinolones is superior to standard drug dosing on the intensive care. The two study groups are defined as 1) the intervention group, which will receive TDM of study antibiotics, and 2) the control group, which will receive treatment as usual. The trial is anticipated to include 450 patients from over 8 ICUs in the Netherlands over a 24 month period. Data analysis will be done based on the intention-to-treat principle.

Patients will be randomised to one of the study groups by a 1:1 ratio, assigned by a computerised randomisation programme (ALEA Randomisation Service). The block randomisation is stratified by study centre and antibiotic group.

Study antibiotics are cefotaxime, ceftazidime, ceftriaxone, cefuroxime, amoxicillin, amoxicillin with clavulanic acid, flucloxacillin, piperacillin with tazobactam, meropenem and ciprofloxacin.

### Participants

All patients admitted to the ICU wards and given standard of care intravenous therapy of the study antibiotics will be screened against the inclusion criteria. Identification of eligible patients will occur on a daily basis by training research or clinical staff at the participating study sites. Informed consent is obtained before participation in the trial by the research staff or responsible clinician. If a patient is incapable of giving consent, a legal representative will be inquired. If possible, informed consent from the patient is obtained at day five in case of deferred consent by a legal representative.

### Inclusion and exclusion criteria

Patients will need to be 18 years or older, receive intravenous antibiotic therapy of the study antibiotics and treatment should be aimed for at least 2 days at time of inclusion. Patients will be excluded in the case of pregnancy, antibiotic cessation before the first blood sample collection, already being enrolled in this trial or any other intervention trial, or receiving study antibiotics only as prophylaxis within the context of selective digestive tract decontamination (SDD). Medium care and burn wound patients will also be excluded. Patients will need to fulfil all the inclusion and none of the exclusion criteria at randomisation.

An inclusion scheme is followed (Appendix 2), in which per hospital and time period the anticipated inclusion rate is presented.

### Sample size calculation

We hypothesized that active TDM versus non-TDM will decrease median ICU LOS from 7 to 6 days (baseline 7 ± 3.5, data of five hospitals ICU LOS [28, 29]). With alpha level of 0.05, and power of 0.80, the sample size is calculated as 192 per group. Considering a drop-out percentage of approximate 15%, 450 patients are required in total.

### Pharmacokinetic sampling

**Fig. 1:**
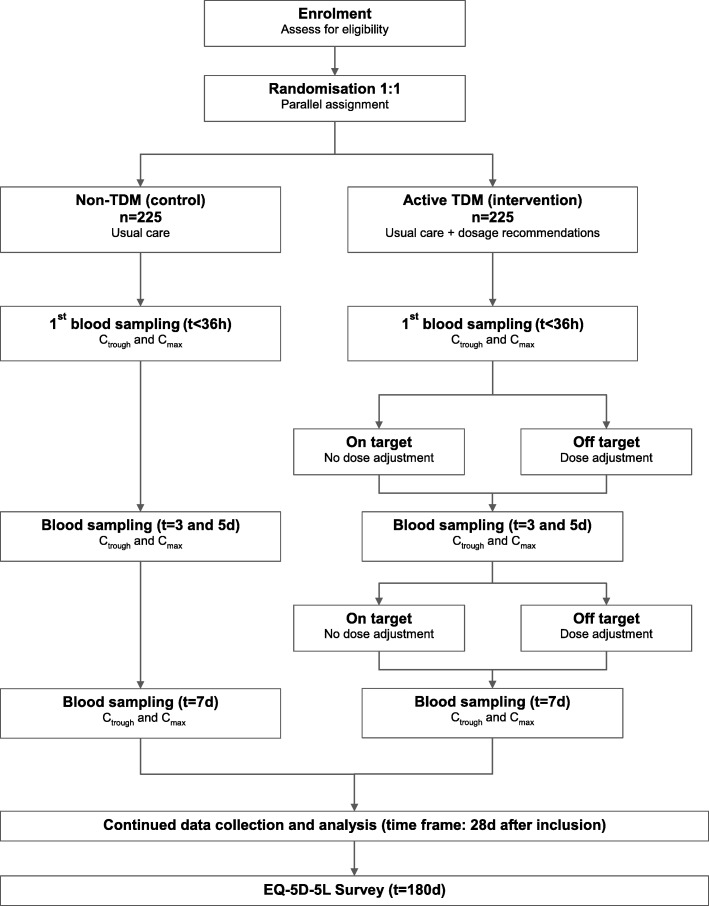
Diagram of the trial design. C_trough_, trough concentration of study antibiotic; C_max_, maximum concentration of study antibiotic

Blood samples will be obtained from the patient at day 1, 3, 5 and 7 (Fig. 1) during the morning round of antibiotic administration. A C_trough_ (30 min before antibiotics infusion) and C_max_ (30 min after completion of antibiotics infusion) will be collected for each measuring moment. Total and unbound drug concentrations will be measured in serum by means of a validated LC-MS/MS method in the Erasmus University Medical Center (Erasmus MC) [30]. Samples obtained in an external centre will be transported to the Erasmus MC for analysis. In the intervention group the analysis will be performed and reported on the same day. In the control group blood samples will be collected according to the same sampling scheme, and the samples will be analysed in bulk later.

### Modelling

Patient-specific parameters and antibiotic serum levels will be used to calculated expected antibiotic exposures. InsightRX™ (version 1.15.16, San Francisco, California), a cloud-based clinical decision support platform, will be used to assess individualized dosing regimens using model-informed precision dosing. For model fitting and simulation of concentration time courses, validated and peer-reviewed research of population-based PK/PD models in ICU patients will be used. Based upon these models and the serum antibiotic levels, time unbound levels above MIC (fT > MIC), unbound area under the curve divided by MIC (fAUC_0-24h_/MIC) and through concentrations (C_trough_) will be calculated. Dose adjustment in the intervention group is performed based on the PK/PD targets and dose reduction thresholds as described in Table 1. For each of the antibiotics, the ECOFF of the presumed pathogen, as defined by the European Committee on Antimicrobial Susceptibility Testing (EUCAST), was used [31].

**Table 1:** PK/PD targets and thresholds for dose reduction of antibiotic groups

Antimicrobials	Target	Threshold dose reduction
Beta-lactams	fT above MIC = 100%	C_trough_ > 10 x MIC
Ciprofloxacin	fAUC_0-24h_/MIC ratio ≥> 100	fAUC_0-24h_/MIC ratio > 400

### Trial intervention

Based upon the abovementioned calculations, a dosage recommendation will be communicated on the same day as sampling to the treating physician by a hospital pharmacist or trained researcher. In case of under- or overdosing, the dosage will be increased or decreased as described in Table 2. Adherence or deviation from this advice will be registered in the electronic Case Report File (eCRF).

**Table 2:** Dosage advice options for intervention

Dosage adjustment	Dose recommendations
Dose increase	Increase dose administration frequency by 25–50%
	Increase dose by 25–50% with same dosing frequency
Dose reduction	Decrease dose administration frequency
	Decrease dose by 25–50% with same dosing frequency
	Withhold administration for 1 day

### Data collection

All collected data will be stored into an eCRF. Laboratory data will include: serum liver enzymes, bilirubin, creatinine, C-reactive protein, procalcitonin, haemoglobin, white blood cells, albumin and thrombocytes. Clinical data involve the daily Sequential Organ Failure Assessment (SOFA) score, fluid balance, Acute Physiology and Chronic Health Evaluation version 4 (APACHE IV) score, surgery in the 5 days before admission, use of extra-corporal devices, mechanical ventilation, other antibiotics next to the study antibiotics and comorbidities. We will also collect the admission data which includes the admission diagnosis and reason for starting antibiotics, admission and discharge dates and 28-day mortality. The most prevalent and most severe side effects will also be collected. Quality of life will be assessed at 6 months with EuroQol™ 5D-5 L Questionnaire. The economic evaluation will be performed from a hospital perspective. Only direct medical costs will be included. We will use charges as published in Dutch guidelines as a proxy of real costs.

### Statistical analysis

#### Baseline characteristics

We will include baseline characteristics related to the patient, the admission, and hospital. A complete overview of variables measured in the DOLPHIN trial is presented in Appendix 3. Continuous variables will be presented as mean with corresponding standard deviation (SD) if normally distributed, and median with ranges if data are skewed. Normality will be assessed using the Shapiro-Wilk test. Categorical variables will be given in numbers and percentages. We will assess whether these baseline characteristics are significantly different between the two study groups. For continuous variables we will compare the means using an independent-sample t-test or Mann Whitney-U test when normally or non-normally distributed, respectively. For categorical variables, we will examine statistical differences between study groups using a chi-square test.

#### Primary outcome

The primary outcome is ICU LOS. This outcome is based on count data which will be analysed using a poisson regression. The ICU LOS of patients transferred to another ICU are calculated between ICU admission and transfer date. The effect size will be expressed in a crude relative risk estimate and absolute risk reduction.

Next to the crude study effect, control variables will be added to the poisson regression model in case baseline characteristics are statistically significant different with a *P*-value < 0.15 in the univariate analysis. This sensitivity analysis is performed to adjust for residual (large) baseline imbalances to assess their impact and to assess the robustness of the primary analysis.

#### Secondary outcomes

We identified eight secondary outcomes, namely: (1) ICU survival; (2) 28 day survival; (3) incidence of most common side-effects. Regarding the effect of the treatment: (4) antibiotic target attainment; (5) sickness severity change with delta-SOFA scores between start of antibiotics and day 5 [32]; (6) changes in infectious parameters; (7) quality of Life 6 months after admission with EuroQol™ 5D-5 L Questionnaire; (8) Costs and cost-effectiveness from a hospital perspective.

Statistically significant differences for continuous and categorical variables between study groups will be assessed using an independent sample t-test and chi-square test, respectively. If a continuous variable is non-normally distributed, a Mann Whitney-U test will be employed to assess the statistical differences. In case of imbalances between the two groups (as assessed by the univariate analyses), we will switch to poisson regression, binary logistic regression, or linear regression for count, binary outcome, or continuous outcomes, respectively. We will adjust these models for the imbalanced variables.

The cost-effectiveness of TDM will be assessed by calculating the incremental cost-effectiveness ratio, defined as the difference in costs of TDM compared to usual care, divided by the average change in effectiveness.

### Data monitoring

Because of the nature of the trial with a low risk of intermediate complications, an independent monitor will visit each study site every 6 months. 25% of all cases are randomly selected for verification by the independent monitor. Informed consent, source data and reported serious adverse events (SAEs) are reviewed for errors. The data will be pseudonymised when stored in the database and then used for analysis.

### Serious adverse events

SAEs will be reported to the local medical ethics committee through an online platform within 7 days of occurrence. These will include deaths or re-admissions within the trial period of 6 months follow-up. Selected adverse drug reactions are reported until day seven. Research staff is trained how to address SAEs and how to report these to the coordinating researcher.

### Dissemination

Findings will be submitted to peer-reviewed journals for publication, and to local and international conferences. As we have multiple secondary outcomes, we expect to submit multiple publications to peer-reviewed journals. Findings will be communicated to the public through media coverage and personal website(s).

## Discussion

It is important to dose antibiotics correctly to prevent therapeutic failure, toxicity, and antimicrobial resistance. The DOLPHIN trial, a multi-centre RCT with clinical outcome as an endpoint, aims to answer the question whether TDM of beta-lactam and fluoroquinolones in critically ill patients is of added value. This design is quite unique in TDM studies. Several studies have retrospectively reported a better outcome when beta-lactam pharmacodynamic targets are attained. However, these positive effects have never been confirmed in a prospective clinical trial.

Continuous infusion of antibiotics is being used in an increasing number of ICUs. The results seem promising on clinical cure rate and ventilator-free days [33, 34]. However - with one dose for all patients - it still does not take the augmented renal clearance and variability of ICU patients’ pharmacokinetics into account.

TDM is already gaining terrain in guidelines and reviews, in which TDM of beta-lactam antibiotics is advised when high PK variability is expected [35, 36]. Nonetheless, these guidelines are not based upon prospective randomised trials.

Patient recruitment is an ongoing challenge in many RCTs. We will evaluate the inclusion rate at multiple time points during the trial. If the inclusion rate is too low, we will approach additional centres to participate in the trial.

Until now, the effect of early TDM of beta-lactam and fluoroquinolones on clinical outcome in the critically ill has not yet been investigated in a multi-centre RCT. This makes the DOLPHIN trial unique in its field. Its findings may lead to new insights and more evidence based clinical management of the patient receiving antibiotics on the ICU.

## Figures and Tables

**Fig. 2:**
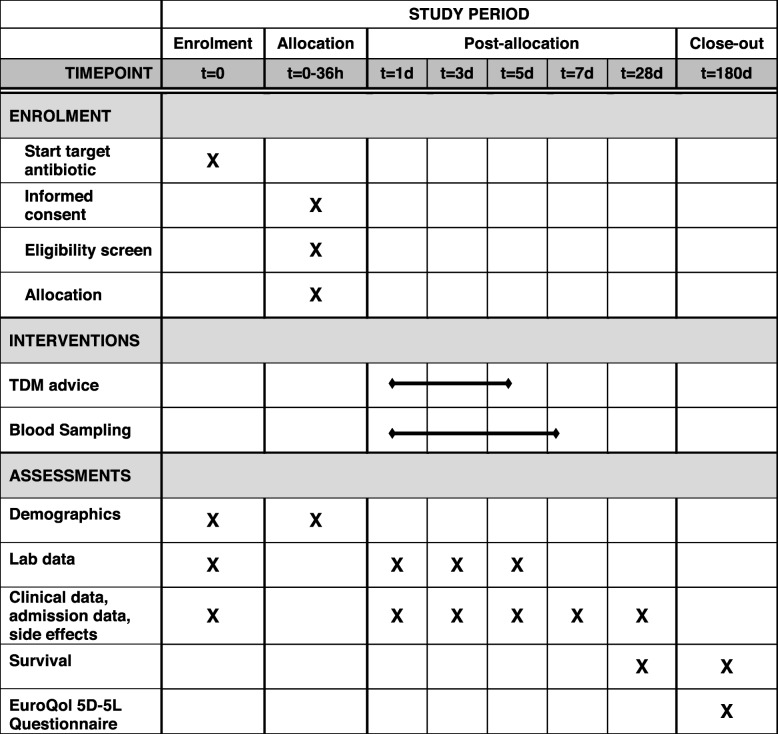
SPIRIT Figure of the trial procedure timeline. The SPIRIT figure of the DOLPHIN trial. The time path of the enrolment, intervention and assessments in the trial

## Data Availability

The datasets used and/or analysed during the current trial are available from the corresponding author on reasonable request after publication. The data will need to be requested in the context of research approved by a medical ethical committee and will need to follow the General Data Protection Regulation.

## Appendix 2

**Table 3:** Participating medical centres and the inclusion estimations. The participating centres of the DOLPHIN trial, the date of the start of the study and the estimated inclusions in study sites

Study sites	Start date	Expected enrollment
Erasmus Medical Center (Rotterdam)	01–11–2018	100
Haaglanden Medical Center (The Hague)	01-07-2019	50
Haga Hospital (The Hague)	01-07-2019	50
Franciscus Gasthuis en Vlietland (Rotterdam)	01-07-2019	50
Amphia Hospital (Breda)	01-10-2019	50
Groene Hart Hospital (Gouda)	01-12-2019	50
Isala Hospital (Zwolle)	01-12-2019	50
Diakonessenhuis (Utrecht)	01–01–2020	50

## Appendix 3

List of variables measured in the Dolphin trial. This appendix contains the list of variables measured in the Dolphin trial.

**Demographic Data**

- Age
- Sex
- Height
- Weight
- Hospital

**Clinical data**

- ICU admission diagnosis
- Indication for antibiotic therapy
- Comorbidities (Charlson comorbidity index)
- Illness severity scores (APACHE and SOFA)
- Sepsis III criteria
- Daily maximum body temperature
- Presence of extracorporeal circuits (e.g. RRT (renal replacement therapy), ECMO (extracorporeal membrane oxygenation))
- Surgery before admission
- ICU mortality
- Hospital mortality
- 6-month mortality
- ICU length of stay
- Hospital length of stay
- Fluid balance
- Most common side effects

**Clinical chemistry data**

- Kidney related: Creatinine, and Urea
- Liver related: Albumin, ASAT, ALAT, GGT, ALP, and Total bilirubin
- Infectious related: CRP, Procalcitonin, and White blood cell count
- Hematological related: Hemoglobin, Trombocytes, and aPTT

**Antibiotic dosing data**

- All antibiotic use during 28 day period (target antibiotic and additional antibiotics)
- Start and end dates
- Initial dose and frequency
- Adjustments to dose and frequency of target antibiotic
- Time of sampling and antibiotic administration

**Other**

- Known or presumed pathogen (positive blood culture and organisms isolated)
- Bacterial susceptibility breakpoints: Minimum Inhibitory Concentration (MIC)
- Quality of life (EQ-5D-5 L)

## Abbreviations

APACHE IV: Acute Physiology and Chronic Health Evaluation IV
AUC_0-24h_: Area under the time-concentration curve up to 24 h
AUC_0-24h_/MIC: Area under the curve divided by MIC
C_max_: Maximum concentration
CRF: Case report file
C_trough_: Trough concentration
ECOFF: Epidemiologic cut-off values of the European Committee on Antimicrobial Susceptibility Testing
eCRF: Electronic case report file
eGFR: Estimated glomerular filtration rate
EuroQol: 5D- 5 L 5 level EuroQol 5- Dimensions questionnaire
fAUC_0-24h_/MIC: Unbound drug level area under the curve divided by MIC
fT > MIC: Unbound drug level time above MIC
ICU: Intensive care unit
ICU LOS: ICU length of stay
LC-MS/MS: Liquid chromatography–mass spectrometry with second mass spectrometry
MIC: Minimum inhibitory concentration
PD: Pharmacodynamic
PDT: Pharmacodynamic target
PK: Pharmacokinetic
PK/PD: Pharmacokinetic/pharmacodynamic
RCT: Randomised controlled trial
SAEs: Serious adverse events
SD: Standard deviation
SDD: Selective Digestive tract Decontamination
SOFA: Sequential Organ Failure Assessment
T > MIC: Time above MIC
TDM: Therapeutic drug monitoring
